# Traditional Chinese Medicine *Yang-Gan-Wan* Alleviated Experimental Hepatic Damage by Inhibiting Oxidation, Inflammation, and Apoptosis in Cell and Mouse Models

**DOI:** 10.1155/2021/2556352

**Published:** 2021-10-05

**Authors:** Chia-Wen Yeh, Wan-Jhen Wu, Chen-Wen Lu, Sheue-Er Wang, Wu-Chang Chuang, Ming-Chung Lee, Chung-Hsin Wu

**Affiliations:** ^1^School of Life Science, National Taiwan Normal University, Taipei City, Taiwan; ^2^Pathological Department, Saint Paul's Hospital, Taoyuan City, Taiwan; ^3^Sun Ten Pharmaceutical Co., Ltd., New Taipei City 23143, Taiwan; ^4^Brion Research Institute of Taiwan, New Taipei City 23143, Taiwan

## Abstract

A hepatoprotective medicine, *Yang-Gan-Wan* (YGW), was used to treat hepatic damage in cell and mouse models. We performed a 1,1-diphenyl-2- picrylhydrazyl (DPPH) assay and found that YGW exhibited a significantly high free radical scavenging ability. Furthermore, the results of the 3-(4,5-dimethylthiazol-2-yl)-2,5-diphenyltetrazolium bromide (MTT) assay revealed that YGW treatment could alleviate lipopolysaccharide (LPS)-induced damage in Kupffer cells (liver macrophages). Enzyme-linked immunosorbent assay results demonstrated that YGW treatment could alleviate LPS-induced inflammation in Kupffer cells by inhibiting the expression of tumor necrosis factor (TNF)-*α* and interleukin (IL)-1*β*. By quantifying the serum levels of alanine aminotransferase (ALT) and aspartate aminotransferase (AST), we found that YGW treatment could alleviate hepatic damage and improve immunity in acetaminophen- (APAP-) treated mice by inhibiting the expression of ALT and AST. The findings of hematoxylin and eosin and Masson's trichrome staining indicated that YGW treatment could alleviate hepatic damage and reduce collagen fiber formation in the liver tissue of APAP-treated mice. Furthermore, immunohistochemistry staining and Western blot results showed that YGW treatment could alleviate oxidative stress, inflammation, and apoptosis in the liver tissue of APAP-treated mice by enhancing superoxide dismutase 2 (SOD2) expression but inhibiting TNF-*α* and caspase 3 expression. Our results suggest that YGW treatment exerted hepatoprotective effects on LPS-treated Kupffer cells and APAP-treated mice by inhibiting oxidation, inflammation, and apoptosis.

## 1. Introduction

Liver diseases are generally silent. When hepatic symptoms appear, the hepatic damage is usually considerably severe because the liver contains no nerves and thus does not transmit pain signals. With human and economic development, work pressure and excessive alcohol consumption often cause hepatic damage and diseases. Hepatic diseases, such as hepatic steatosis, fatty liver, hepatitis, liver fibrosis, cirrhosis, and liver cancer, can adversely affect human health and exhibit different symptoms. Hepatic fibrosis is mainly caused by a chronic hepatitis-related viral infection and obesity-related fatty liver. Hepatic cirrhosis occurs in the later stage of hepatic fibrosis and can increase the risk of hepatic failure and primary liver cancer [1]. However, research on the treatment of hepatic damage and diseases is substantially limited.

Currently, no drugs are available to treat hepatic fibrosis. Liver transplantation is the only treatment available. Developing new therapies that can safely and effectively prevent or reverse hepatic damage is crucial. The use of natural products and herbs for treating hepatic fibrosis and damage has garnered increased attention because of the various biological activities of these natural medicines. Hepatic cell and mouse models can be used to investigate the hepatoprotective effect of traditional Chinese medicine (TCM) preparations on chemical-, immune-, alcohol-, and drug-induced hepatic injury. Many TCM preparations can effectively alleviate hepatic damage and diseases [2]. In recent years, there has been a research trend in the development of Chinese herbal medicines. Due to their wide availability, low toxicity, and low side effects, past studies such as Sho-saiko-to and *Salvia miltiorrhiza* have a long history of clinical treatment of liver disease [3–6], but there is a lack of scientific data to support the investigation of the efficacy of Chinese medicine ingredients, and the mechanism of action is unknown.

*Yan-Gan-Wan* (YGW) is among the most widely used TMC preparations and can effectively alleviate liver damage. In mice treated with allyl alcohol, acetaminophen, and carbon tetrachloride, YGW treatment could effectively alleviate hepatic damage [7]. In addition, YGW exerted protective effects against hepatic apoptosis in mice treated with anti-Fas antibodies [8]. Furthermore, YGW treatment could prevent and reverse the activation of hepatic stellate cells that underwent fibroblast transdifferentiation to participate in liver fibrosis [9]. The findings of a chromatographic fingerprint analysis revealed that polyphenolic rosmarinic acid and baicalin, the two active phytocompounds present in YGW, could inhibit the activation of hepatic stellate cells and the progression of hepatic fibrosis in mice with cholestatic hepatic fibrosis [10]. Hepatotoxicity is the main cause of death in patients with acute liver failure. Excessive exposure to chemical agents, immunological agents, and drugs as well as high alcohol consumption may lead to liver damage due to reactive oxygen species (ROS) production, oxidative stress, inflammation, and apoptosis, resulting in hepatotoxicity. Although many studies have reported that YGW treatment exerted hepatoprotective effects on cell and mouse models of hepatic injury [7–10], whether YGW treatment can alleviate hepatic damage through antioxidative, anti-inflammatory, and antiapoptotic activities remains unclear.

The present study investigated the beneficial effects of YGW treatment against hepatotoxicity in lipopolysaccharide- (LPS-) treated Kupffer cells and acetaminophen- (APAP-) treated mice. Our results revealed that YGW treatment exerted hepatoprotective effects on LPS-treated Kupffer cells and APAP-treated mice by inhibiting oxidation, inflammation, and apoptosis.

## 2. Materials and Methods

### 2.1. Preparation of YGW

In this study, YGW was obtained from Sun Ten Pharmaceutical Company, New Taipei City, Taiwan. Each 600 mg sample of YGW contained Radix *Angelicae Sinensis* (41 mg), Semen *Plantaginis* (41 mg), Radix *Paeoniae Alba* (41 mg), Radix *Saposhnikoviae* (41 mg), Nux *Prinsepiae* (41 mg), Radix *Rehmanniae Preparata* (41 mg), Rhizoma *Chuanxiong* (41 mg), Fructus *Aurantii Immaturus* (41 mg), and honey (272 mg). A chromatographic fingerprint analysis of YGW was performed using high-performance liquid chromatography (HPLC).

### 2.2. DPPH Assay of YGW

The antioxidative activity of YGW was assessed by performing a 1,1-diphenyl-2-picrylhydrazyl (DPPH) (D9132, Sigma-Aldrich, St. Louis, MO, USA) assay. The DPPH assay procedure used in this study was similar to a previous study [11]. The DPPH radical scavenging activity of YGW was analyzed and quantified using a mixture of DPPH in methanol.

### 2.3. Cytotoxicity Assay of Kupffer Cells Treated with YGW

The cytotoxicity of Kupffer cells treated with YGW was assessed using a 3-(4,5-dimethylthiazol- 2-yl)-2,5-diphenyltetrazolium bromide (MTT) (M5655, Sigma-Aldrich) assay. Kupffer cells (SCC119) were obtained from Sigma-Aldrich. The method used to culture Kupffer cells was similar to a previous study [12]. For the study of liver injury, function, and mechanism, we used Immortalized Kupffer cells (KC) to treat YGW and LPS in vitro model. Kupffer cells were cultured in a 1 : 1 mixture of RPMI Medium and Pri-Grow II medium (ABM-TM002) with 10% fetal bovine serum (Thermo Fisher Scientific™), 100 *μ*g/mL streptomycin, and 100 U/mL penicillin. Kupffer cell culture medium was replaced every 72 hours, and cells were grown at 37°C in the presence of 5% CO_2_. Kupffer cell culture was approved under the approval of the Biological Experimental Safety Committee of the National Taiwan Normal University. After the treatment of hepatic Kupffer cells with YGW for 24 h, the viability of such cells was analyzed and quantified using the MTT assay with or without LPS (L-2654, Sigma-Aldrich) treatment.

### 2.4. Inflammatory Cytokine Assay of Kupffer Cells Treated with YGW

The presence of inflammatory cytokines in Kupffer cells treated with YGW was examined using the enzyme-linked immunosorbent assay (ELISA). Similar to our previous study [13], purified rat anti-mouse tumor necrosis factor-*α* (TNF-*α*) (88–7324; Invitrogen, Carlsbad, CA, USA) and rat anti-mouse interleukin-1*β* (IL-1*β*) (88–7013; Invitrogen) were added to a 96-well microwell plate (Thermo Scientific Nunc, Nunc AS, Copenhagen, Denmark). After the plate was washed three times with PBST buffer, the cell supernatant sample and antibody standard were added. Subsequently, biotin-conjugated anti-mouse TNF-*α* and IL-1*β* antibodies were added to the 96-well microwell plate and maintained there for 1 h at room temperature. After the plate was washed five times with PBST buffer, we added avidin-linked peroxidase (avidin–horseradish peroxidase conjugation) to it at room temperature and maintained it there for 30 min. After the plate was washed six times with PBST buffer, we added the substrate (tetramethylbenzidine) to it at room temperature and maintained it there for 30 min. The reaction was terminated by the addition of 2% H_2_SO_4_, and the absorbance value was 450–570 nm.

### 2.5. Animal Preparation

Male Institute of Cancer Research (ICR) mice were obtained from BioLASCO Breeding Center (Yilan, Taiwan) and then randomly divided into six groups: sham, APAP (acetaminophen, Sigma-Aldrich Co., St. Louis, MO, USA) treatment, APAP treatment after YGW feeding (APAP + pre-YGW), APAP treatment before YGW feeding (APAP + post-YGW), APAP treatment before and after YGW feeding (APAP + pre- and post-YGW), and APAP treatment after N-acetyl-L- cysteine (NAC, Sigma-Aldrich Co.) feeding (APAP + NAC). The mice in the sham group were fed only dimethyl sulfoxide twice daily for 14 days. The mice in the APAP group were administered only intraperitoneal APAP injections (10 mg/kg body weight). The mice in the APAP + pre-YGW group were administered intraperitoneal APAP injections (400 mg/kg body weight, IP) after YGW feeding (100 mg/kg, pH close to 7.0, oral gavage) through their drinking water once daily for 7 days. The mice in the APAP + post-YGW group were administered intraperitoneal APAP injections (400 mg/kg body weight, IP) before YGW feeding (100 mg/kg, pH close to 7.0) through their drinking water once daily for 7 days. The mice in the APAP + pre- and post-YGW group were administered intraperitoneal APAP injections (400 mg/kg body weight, IP) before and after YGW feeding (100 mg/kg, pH close to 7.0) through their drinking water once daily for 14 days. The mice in the APAP + NAC group were administered intraperitoneal APAP injections (400 mg/kg body weight, IP) after NAC feeding (600 mg/kg, pH close to 7.0, oral gavage) through their drinking water twice a week. Our animal experiments followed the 3R principles and were performed in accordance with international guidelines for the Care and Use of Laboratory Animals of National Taiwan Normal University (Permit number: NTNU/Animal Use/No. 109028/March 31, 2020).

### 2.6. Blood Glutamic Oxaloacetic Transaminase and Glutamic Pyruvic Transaminase Analysis

In this study, blood alanine aminotransferase (ALT) and aspartate aminotransferase (AST) levels were measured using ELISA. Similar to a procedure in our previous study [14], after the animal experiments, the mice were anesthetized and then killed. Blood samples were collected in a 2 mL centrifuge tube. After centrifuging the sample at 3,000 ×g at 4°C for 15 min, we collected the supernatant and stored it at −80°C until analysis. Serum AST and ALT levels were measured by using their analysis kits (Invitrogen), respectively, and analyzed by using an automatic biochemical analyzer (Spotchem EZ SP 4430, ARKRAY, Kyoto, Japan). ELISA was performed according to the manufacturer's instructions.

### 2.7. Liver Tissue Staining

In this study, liver tissue was examined through hematoxylin and eosin (H&E), Masson's trichrome, and immunohistochemistry (IHC) staining. Similar to our previous study approach [14], we surgically collected liver tissue specimens, embedded them in paraffin, cut them into 5 *μ*m thick sections, and mounted them on slides. An H&E polymer detection system (Leica Biosystems Newcastle Ltd. Buffalo Grove, IL, United States) was used to examine liver cells, and Masson's trichrome staining kit (Artisan Masson's trichrome staining kit, Agilent, Santa Clara, CA) was used to evaluate collagen fiber formation. The IHC staining procedure followed in this study was similar to that used in our previous study [15]. Antibodies against superoxide dismutase 2 (SOD2), TNF-*α*, and caspase 3 (Cell Signaling Technology, Danvers, MA, USA) were, respectively, used in the liver tissue specimens to determine the expression of SOD2, TNF-*α*, and caspase 3.

### 2.8. Western Blot Analysis

In this study, the protein expression of SOD2, TNF-*α*, and caspase 3 in the liver tissue was assessed through Western blotting. Similar to our previous study [13–16], we homogenized and quantified liver tissues and then transferred them onto polyvinylidene difluoride membranes (GE Healthcare Life Sciences, Barrington, IL, USA). Antibodies against *β*-actin (Thermo Fisher Scientific), SOD2, TNF-*α*, and caspase 3 (Cell Signaling Technology) were detected, visualized, and quantified using ImageJ analysis software (version 1.48t, NIH, Wayne Rasband, Washington DC, USA).

### 2.9. Statistical Analysis

All experiments were repeated at least three times in each group, and the results were expressed as mean ± standard error of the mean (SEM). We used one-way analysis of variance followed by the Student–Newman–Keuls multiple comparison posttest, for statistical analyses. Differences between the groups were considered to be significant when the *P* value was at least 0.05.

## 3. Results

### 3.1. Chromatographic Fingerprint Analysis of YGW

**Figure 1:**
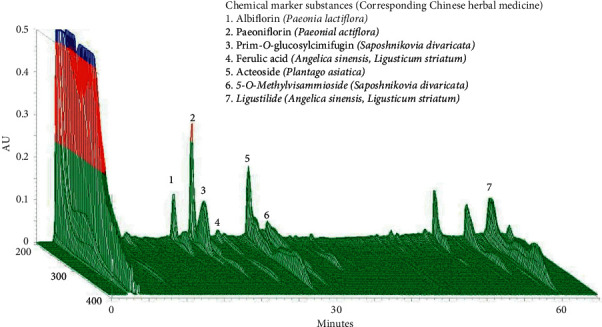
3D chromatographic fingerprint analysis of the hepatoprotective medicine YGW by using 3D HPLC. The following bioactive marker substances were identified: albiflorin and paeoniflorin from *Paeonia lactiflora*, prim-O-glucosylcimifugin and 5-O-methylvisammioside from *Saposhnikovia divaricate*, acteoside from *Plantago asiatica*, and ferulic acid and ligustilide from *Angelica sinensis* and *Ligusticum striatum*. AU, arbitrary perfusion units.

Figure 1 shows three-dimensional (3D) chromatographic fingerprint analysis of YGW performed using 3D HPLC. The following bioactive marker substances were identified: albiflorin and paeoniflorin from *Paeonia lactiflora*, prim-O-glucosylcimifugin and 5-O-methylvisammioside from *Saposhnikovia divaricata*, acteoside from *Plantago asiatica*, and ferulic acid and ligustilide from *A. sinensis* and *Ligusticum striatum*.

### 3.2. Antioxidative Capacity of YGW

**Figure 2:**
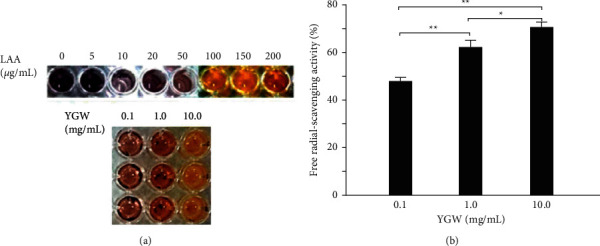
Antioxidative capacity of YGW. (a) DPPH assay using various concentrations of YGW treatment for 30 minutes. L-ascorbic acid is a standard antioxidative compound. (b) The DPPH free radical scavenging activity of YGW significantly increased with their concentration (*n* = 3 for each group; values are presented as mean ± SEM, ^*∗∗*^*P* < 0.01, ^*∗*^*P* < 0.05, one-way ANOVA followed by the Student–Newman–Keuls multiple comparison post hoc test).

Figure 2(a) shows the antioxidative capacity of YGW determined using the DPPH assay. L-ascorbic acid is a standard antioxidant compound. As shown in Figure 2(b), the DPPH free radical scavenging activity was 48%, 62%, and 70% for 0.1, 1.0, and 10.0 mg/mL YGW, respectively. We observed that the free radical scavenging activity significantly increased with YGW concentration (*P* < 0.01–0.05). Our results suggested that YGW exhibits a high antioxidative capacity to scavenge free radicals.

### 3.3. YGW Treatment Alleviates the Cytotoxicity of LPS-Treated Kupffer Cells

**Figure 3:**
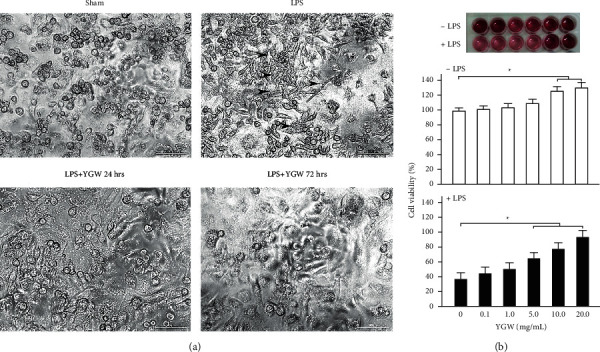
YGW treatments effectively alleviated LPS-induced damage in Kupffer cells. (a) Damaged and elongated Kupffer cells (indicated by the arrow) were observed after treatment with 1 *μ*g/mL LPS; their morphology was restored to normal after treatment with 15 mg/mL YGW for 24 and 72 h scale bar = 30 *μ*m. (b) MTT assay showed the cell viability of liver Kupffer cells under different concentrations of YGW. Irrespective of LPS treatment, the viability of Kupffer cells treated with YGW (5–20 mg/mL) significantly increased compared with that of Kupffer cells not treated with YGW (*n* = 3 for each group; values are presented as mean ± SEM).

Figure 3(a) shows the potential cytotoxic effect of YGW treatment on hepatic Kupffer cells that was measured using an MTT assay. Hepatic Kupffer cells that were damaged and elongated after treatment with <1 *μ*g/mL LPS had partly restored normal morphology after treatment with 15 mg/mL YGW for 24 or 72 h (Figure 3(a)). The viability of Kupffer cells was 100%, 101%, 104%, 108%, 126%, and 130% after treatment with 0.1, 1.0, 5.0, 10.0, and 20.0 mg/mL YGW, respectively (Figure 3(b)). We observed that the viability of Kupffer cells treated with 10–20 mg/mL YGW was significantly higher than that of Kupffer cells not treated with YGW (*P* < 0.05). As shown in Figure 3(b), the viability of LPS-treated hepatic Kupffer cells was 38%, 44%, 51%, 65%, 77%, and 94% after treatment with 0.1, 1.0, 5.0, 10.0, and 20.0 mg/mL YGW, respectively. After LPS-induced damage, the viability of hepatic Kupffer cells decreased significantly (*P* < 0.01). We found that the viability of Kupffer cells treated with 5–20 mg/mL YGW was significantly higher than that of LPS-treated Kupffer cells not treated with YGW (*P* < 0.05). Our results suggest that YGW treatment exerted protective effects on Kupffer cells after LPS-induced damage.

### 3.4. YGW Treatment Alleviates the Inflammation of LPS-Treated Kupffer Cells

**Figure 4:**
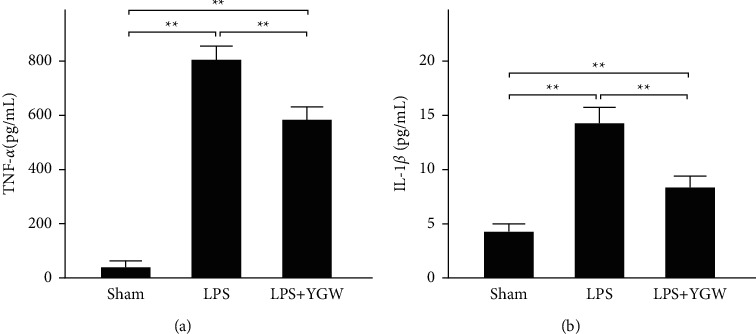
YGW treatment effectively alleviated LPS-induced inflammation in Kupffer cells for 24 hours. The expression of the inflammatory markers TNF-*α* (a) and IL-1*β* (b) in Kupffer cells was significantly increased after LPS treatment but significantly decreased after YGW treatment (*n* = 3 for each group; values are presented as mean ± SEM).

Figure 4 shows the presence of inflammatory markers in Kupffer cells examined through ELISA. The levels of the inflammatory marker TNF-*α* in hepatic Kupffer cells were 45, 802, and 582 pg/mL after sham treatment, LPS-induced damage, and YGW treatment after LPS-induced damage, respectively (Figure 4(a)). The levels of the inflammatory marker IL-1*β* in hepatic Kupffer cells were 4.2, 14.1, and 8.2 pg/mL after sham treatment, LPS-induced damage, and YGW treatment after LPS-induced damage, respectively (Figure 4(b)). We found that LPS treatment significantly increased TNF-*α* and IL-1*β* levels in hepatic Kupffer cells (*P* < 0.01), whereas YGW treatment significantly reduced TNF-*α* and IL-1*β* levels in hepatic Kupffer cells after LPS-induced inflammation (*P* < 0.01). Our results indicate that YGW treatment alleviated inflammation in hepatic Kupffer cells after LPS-induced damage.

### 3.5. YGW Treatment Reduces the Hepatic Inflammation-Related Index of APAP-Treated Mice

**Figure 5:**
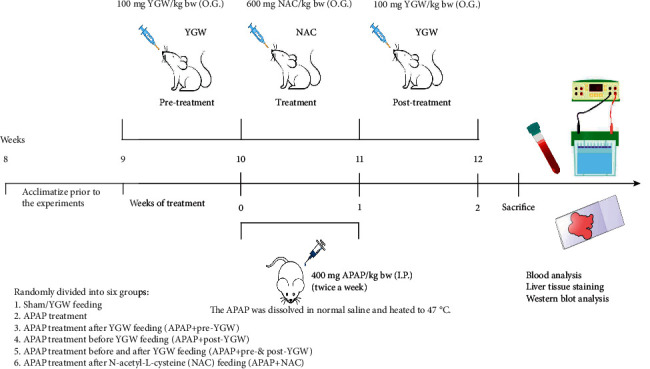
Experimental design that draws the timeline of the action of YGW, LPS, and APAP, and the dosage of drugs and the time points of analyses. The dosage of YGW, APAP, and NAC was also shown. IP: intraperitoneal injection; OG: oral gavage.

**Figure 6:**
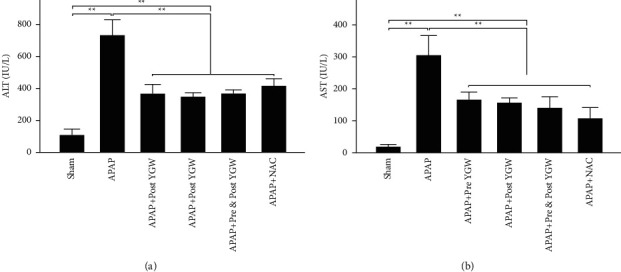
YGW treatment effectively alleviated APAP-induced liver injury in mice. The expression of the liver injury-related markers (a) ALT and (b) AST significantly increased after APAP treatment but significantly decreased in the blood of mice after separating pre-YGW, post-YGW, pre- and post-YGW, and NAC treatments. N-Acetylcysteine (NAC) was the positive control for APAP treatment (*n* = 3 for each group; values are presented as mean ± SEM).

Figure 5 shows the animal experimental design that draws the timeline of the action of YGW, LPS, and APAP and the dosage of drugs and the time points of analyses. Through ELISA, Figure 6 shows the levels of hepatic inflammation-related indices, namely, ALT and AST, which were examined. The ALT levels were 45, 721, 367, 342, 361, and 412 IU/L in the sham, APAP, APAP + pre-YGW, APAP + post-YGW, APAP + pre- and post-YGW, and APAP + NAC groups, respectively (Figure 6(a)). The AST levels were 21, 296, 163, 151, 136, and 105 IU/L in the sham, APAP, APAP + pre-YGW, APAP + post-YGW, APAP + pre- and post-YGW, and APAP + NAC groups, respectively (Figure 6(b)). We found that APAP treatment significantly increased ALT and AST levels in the blood of mice (*P* < 0.01), whereas pre-YGW, post-YGW, pre- and post-YGW, and NAC treatment significantly reduced ALT and AST values in the blood of mice (*P* < 0.01). Our results suggest that YGW treatment could alleviate hepatic inflammation in APAP-treated mice.

### 3.6. YGW Treatment Alleviated Hepatic Injury and Reduced Hepatic Collagen Fiber Formation in APAP-Treated Mice

**Figure 7:**
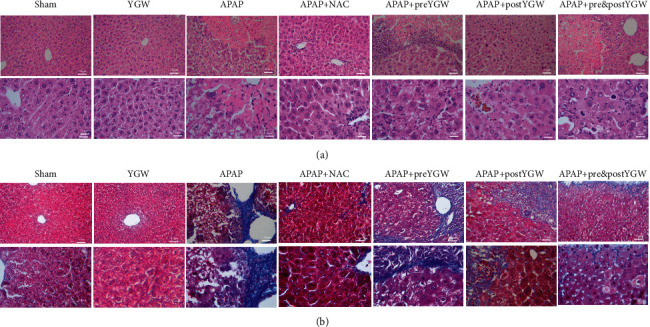
Oral YGW treatment effectively alleviated hepatic damage and reduced collagen fiber formation in APAP-treated mice. (a) H&E staining (200x and 400x) revealed normal liver cells in mice given the sham and YGW treatments but damaged liver cells in mice treated with APAP. After NAC, pre-YGW, post-YGW, and pre- and post-YGW treatment, the damaged liver tissue showed varying degrees of repair. (b) Masson's trichrome staining (200x and 400x) showed collagen fiber formation in the liver tissue of mice treated with APAP (blue color), whereas collagen fiber formation was partly restored in the liver tissue of APAP-treated mice after NAC, pre-YGW, post-YGW, and pre- and post-YGW treatments.

Figure 7 shows H&E staining results of the liver tissue. The liver tissue was complete and healthy in the sham group but extensively damaged in the APAP group. Pre-YGW, post-YGW, pre- and post-YGW, and NAC treatments partially repaired hepatic injury in the liver tissue of APAP-treated mice (Figure 7(a)). Our results of Masson's trichrome staining showed that the liver tissue was complete and healthy in the sham group; however, collagen fiber formation was observed in the liver tissue of the APAP group. Pre-YGW, post-YGW, pre- and post-YGW, and NAC treatments partially reduced collagen fiber formation in the liver tissue of APAP-treated mice (Figure 7(b)). Our results suggest that YGW treatment alleviated hepatic injury and reduced collagen fiber formation in APAP-treated mice.

### 3.7. YGW Treatment Alleviated Hepatic Oxidative Stress in APAP-Treated Mice

**Figure 8:**
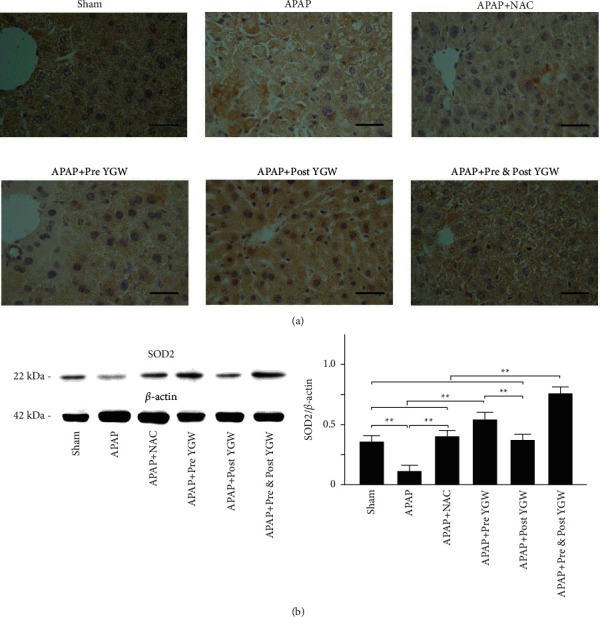
Oral YGW treatment effectively alleviated oxidative stress in the liver tissue of APAP-treated mice. (a) IHC staining showed antioxidant-related SOD2 expression in the liver tissue of sham-treated mice and APAP-treated mice after NAC, pre-YGW, post-YGW, and pre- and post-YGW treatments. Scale bar = 30 *μ*m. (b) SOD2 expression was significantly lower in the liver tissue of mice treated with APAP compared with that in APAP-treated mice after NAC, pre-YGW, post-YGW, and pre- and post-YGW treatments (*n* = 3 for each group; values are presented as mean ± SEM).

Figure 8 shows IHC staining with antioxidant SOD2 in the liver tissue. The results of IHC staining revealed a considerably high expression of the SOD2 in the liver tissue of sham mice; however, decreased SOD2 expression was noted in the liver tissue of the APAP group. Pre-YGW, post-YGW, pre- and post-YGW, and NAC treatments increased SOD2 expression in the liver tissue of APAP-treated mice (Figure 8(a)). Western blot analysis (Figure 8(b)) indicated that the expression of SOD2 relative to that of *β*-actin in the liver tissue was 0.36, 0.11, 0.40, 0.55, 0.37, and 0.75 in the sham, APAP, APAP + pre-YGW, APAP + post-YGW, APAP + pre- and post-YGW, and APAP + NAC groups, respectively (Figure 8(b)). We found that APAP treatment significantly reduced SOD2 expression in the liver tissue of mice (*P* < 0.01), whereas pre-YGW, post-YGW, pre- and post-YGW, and NAC treatment significantly increased SOD2 expression in the liver tissue of APAP-treated mice (*P* < 0.01). Our results indicated that YGW treatment alleviated hepatic oxidative stress in APAP-treated mice.

### 3.8. YGW Treatment Alleviated Hepatic Inflammation in APAP-Treated Mice

**Figure 9:**
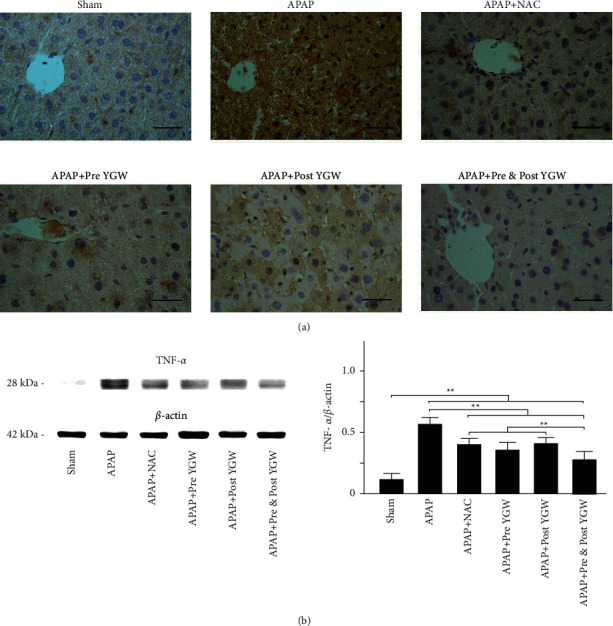
Oral YGW treatment effectively alleviated inflammation in the liver tissue of APAP-treated mice. (a) IHC staining showed the expression of the inflammatory marker TNF-*α* in the liver tissue of sham-treated mice and APAP-treated mice after NAC, pre-YGW, post-YGW, and pre- and post-YGW treatment. Scale bar = 30 *μ*m. (b) TNF-*α* expression was significantly higher in the liver tissue of APAP-treated mice compared with that in APAP-treated mice after NAC, pre-YGW, post-YGW, and pre- and post-YGW treatments (*n* = 3 for each group; values are presented as mean ± SEM).

Figure 9 shows IHC staining with inflammatory marker TNF-*α* in the liver tissue. The IHC staining revealed a considerably low expression of TNF-*α* in the liver tissue of sham group mice; however, increased TNF-*α* expression was noted in the liver tissue of mice in the APAP group. Pre-YGW, post-YGW, pre- and post-YGW, and NAC treatments reduced TNF-*α* expression in the liver tissue of APAP-treated mice (Figure 9(a)). The results of a Western blot showed that the expression of TNF-*α* relative to that of *β*-actin in the liver tissue was 0.12, 0.57, 0.40, 0.36, 0.41, and 0.28 in the sham, APAP, APAP + pre-YGW, APAP + post-YGW, APAP + pre- and post-YGW, and APAP + NAC groups, respectively (Figure 9(b)). We found that APAP treatment significantly increased TNF-*α* expression in the liver tissue of mice (*P* < 0.01), whereas pre-YGW, post-YGW, pre- and post-YGW, and NAC treatment significantly reduced TNF-*α* expression in the liver tissue of APAP-treated mice (*P* < 0.01). Our results demonstrated that YGW treatment alleviated hepatic inflammation in APAP-treated mice.

### 3.9. YGW Treatment Alleviated Hepatic Apoptosis in APAP-Treated Mice

**Figure 10:**
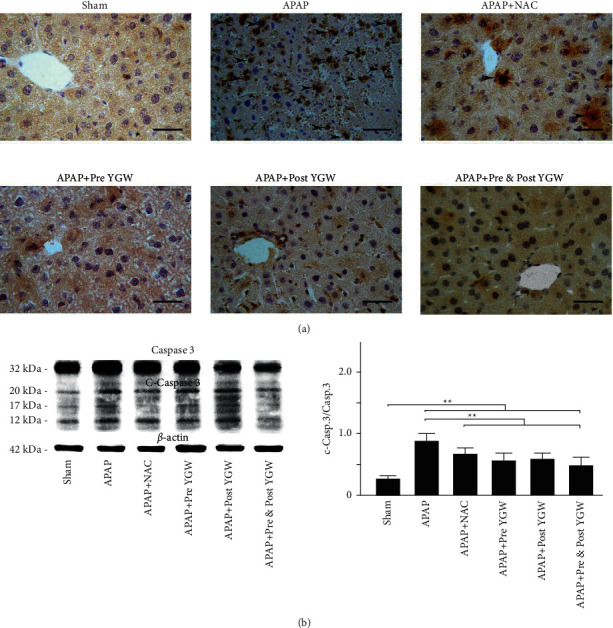
Oral YGW treatment effectively alleviated apoptosis in the liver tissue of APAP-treated mice. (a) IHC staining showed the expression of the apoptosis-related marker c-caspase 3 in the liver tissue of sham-treated mice and APAP-treated mice after NAC, pre-YGW, post-YGW, and pre- and post-YGW treatments. Scale bar = 30 *μ*m. (b) c-caspase 3/caspase 3 expression was significantly higher in the liver tissue of APAP-treated mice than in that of APAP-treated mice after NAC, pre-YGW, post-YGW, and pre- and post-YGW treatments (*n* = 3 for each group; values are presented as mean ± SEM).

Figure 10 shows IHC staining with apoptosis marker cleaved caspase-3 in the liver tissue. The IHC staining results revealed a considerably low expression of the apoptosis marker caspase 3 in the liver tissue of the sham group; however, increased cleaved caspase-3 expression was noted in the liver tissue of the APAP group. Pre-YGW, post-YGW, pre- and post-YGW, and NAC treatments reduced cleaved caspase-3 expression in the liver tissue of APAP-treated mice (Figure 10(a)). The Western blot analysis results showed that the expression of cleaved caspase-3 relative to that of caspase-3 in the liver tissue was 0.28, 0.91, 0.70, 0.59, 0.62, and 0.50 in the sham, APAP, APAP + pre-YGW, APAP + post-YGW, APAP + pre- and post-YGW, and APAP + NAC groups, respectively (Figure 10(b)). We found that APAP treatment significantly increased cleaved caspase-3/caspase-3 expression in the liver tissue of mice (*P* < 0.01), whereas pre-YGW, post-YGW, pre- and post-YGW, and NAC treatments significantly reduced cleaved caspase-3/caspase-3 expression in the liver tissue of APAP-treated mice (*P* < 0.01). Our results indicated that YGW treatment alleviated hepatic apoptosis in APAP-treated mice.

## 4. Discussion

In this study, we used YGW, a hepatoprotective TCM, to treat hepatic injury in cell and mouse models. We performed a DPPH assay to assess the antioxidative capacity of YGW. Our results indicated that YGW exhibited a significantly high free radical scavenging ability (Figure 2). Our study results indicate the potential value of developing more effective TCM preparations that can be used as antioxidative stress-alleviating health foods or therapeutic agents for hepatic diseases. Many natural products exert beneficial effects against hepatic diseases through their antioxidant activities [17]. Oxidative stress plays a vital role in hepatic cirrhosis [18]. Therefore, developing TCM preparations with antioxidative properties for treating the hepatic injury is vital.

Our study demonstrated that traditional Chinese medicine YGW has hepatoprotective effects on LPS-treated Kupffer cells and APAP-treated mice. Although a previous study demonstrates that YGW has protective effects against liver damage induced by allyl alcohol, acetaminophen, or carbon tetrachloride CCl_4_, another study also demonstrates that the protective effects of YGW on CCl_4_ hepatotoxicity are due in part to inhibition of Kupffer cells NF-kappaB activation and TNF-alpha expression by small water-soluble molecules. Through both cell and mouse models of LPS-treated Kupffer cells and APAP-treated mice, our study further demonstrates that the hepatoprotective effects of YGW are inhibiting oxidation, inflammation, and apoptosis.

Kupffer cells often play a key role in early alcoholic hepatic disease. We conducted an MTT assay to examine the cytotoxicity of Kupffer cells treated with YGW. Our results indicated that YGW could alleviate LPS-induced damage in Kupffer cells. In addition, we observed that hepatic Kupffer cells that were damaged and elongated after LPS treatment had restored morphology after YGW treatment (Figure 3). Chronic alcoholism can sensitize Kupffer cells to LPS by enhancing oxidative stress and promoting the secretion of various proinflammatory mediators. Excessive nutrition and chronic alcohol abuse often cause liver diseases such as fatty liver, hepatitis, and cirrhosis [19]. To determine whether YGW treatment can reduce the release of proinflammatory cytokines in Kupffer cells with LPS-induced inflammation, we used ELISA to analyze TNF-*α* and IL-1*β* levels in Kupffer cells. We found that YGW treatment could alleviate LPS-induced inflammation in Kupffer cells by inhibiting the expression of TNF-*α* and IL-1*β* (Figure 4). YGW is considered a hepatoprotective drug due to its anti-inflammatory effect and is often used to treat the hepatic injury caused by excessive alcohol consumption. Hepatic injury can activate Kupffer cells, thus stimulating excessive oxidation and affecting other inflammatory cells (neutrophils and macrophages); this action is accompanied by the release of proinflammatory cytokines such as TNF-*α*, IL-1*β*, and IL-6 [20]. Among such cytokines, TNF-*α* is a major cytokine observed in alcohol-induced hepatic injury [21, 22].

In this study, APAP-induced acute hepatic injury in the mice model was used to examine the effect of YGW on the reduction of hepatic glutathione (GSH) in hepatic injury. AST (also known as aspartate aminotransferase) and ALT (also known as alanine aminotransferase) are enzymes present in liver tissue and are related to the metabolism of amino acids and proteins in the body. When the liver tissue is damaged, AST and ALT may flow into blood, causing hepatic inflammation and damage. Our results revealed that treatment with high APAP concentrations may induce oxidative stress in liver tissue because of an increase in the hepatic function markers AST and ALT. By contrast, YGW treatment significantly reduced hepatic AST and ALT activities in APAP-treated mice (Figure 6). APAP is among the most commonly used analgesics and antipyretics worldwide. However, an overdose of APAP can cause hepatic damage within 24 to 48 h, leading to acute hepatic failure and even death [23–26].

The findings of H&E and Masson's trichrome staining revealed that YGW treatment could alleviate hepatic damage and reduce collagen fiber formation in the liver tissue of APAP-treated mice (Figure 7). Furthermore, the results of IHC staining and a Western blot showed that YGW treatment could alleviate oxidative stress, inflammation, and apoptosis in the liver tissue of APAP-treated mice by enhancing SOD2 expression and inhibiting TNF-*α* and caspase 3 expression (Figures 8–10). These results suggest that YGW and its active ingredients can improve hepatic function; reduce damage caused by oxidative stress, inflammation, and apoptosis; and protect against hepatic damage.

In addition to increasing antioxidant-related SOD2 expression in the liver tissue after YGW treatments (Figure 8), we also found that the DPPH free radical scavenging activity was significantly increased after YGW treatments (Figure 2). Our study indicates that YGW treatments can scavenge free radicals and enhance SOD2 expression in the liver tissue of APAP-treated mice. The antioxidant enzymes such as SOD2 convert dangerous oxidative products to hydrogen peroxide (H_2_O_2_) and then to water because the metabolic mechanism of APAP does affect the lipid oxidation of GSH. After finding that YGW treatment affects the performance of upstream SOD2, then a series of effects will affect the later stage of glutathione peroxidase (GSHPx) metabolic activity that plays a fundamental role in reducing free radicals.

Through the cell and mouse models of LPS-treated Kupffer cells and APAP-treated mice, our study double confirmed that YGW has hepatoprotective effects by inhibiting oxidant stress (Figures 4 and 9). During the invasion of cells by pathogens, pathogen-associated molecules, such as lipopolysaccharides (LPS), initiates an inflammatory response. Through this process, cytokines including TNF-*α* and IL-1*β* play a vital role in normalizing ROS levels. As previous studies have shown an inhibitory effect of YGW on cytochrome P450 enzymes, pre- or cotreatment of YGW requires that the metabolic activation of APAP [9]. Their data showed the protective effects of YGW on hepatotoxicity are due in part to inhibition of NF-*κ*B activation and TNF-*α* expression that may also be related to the suppressed hepatic expression of cytochrome P450 enzymes. From our study, we have shown that pre- (APAP + pre-YGW) or cotreatment (APAP + pre- and post-YGW) of YGW has hepatoprotective effects through inhibiting TNF-*α* expression in the liver tissue of APAP-treated mice (Figure 9).

Gujral et al. [27] A direct comparison between apoptosis and APAP-induced cell death showed very clearly that APAP-induced liver injury is caused by necrosis and not apoptosis. Although necrosis is recognized as the main mode of cell death induced by APAP overdose in animals and humans, more recently an increasing number of publications, especially in the herbal medicine and dietary supplement field, claim an important contribution of apoptotic cell death in the pathophysiology of APAP-induced liver injury. From our study, we just provided IHC staining and evidence that apoptotic marker of cleaved caspase-3/caspase 3 expression was significantly higher in the liver tissue of APAP-treated mice than in those of APAP-treated mice after YGW treatments (Figure 10).

A study reported that 90% of ingested APAP is metabolized in the liver and then excreted in urine; therefore, less than 10% of ingested APAP is converted into N-acetyl-P-benzoquinone imine (NAPQI) that is metabolized into a metabolite through glutathione (GSH). When GSH is depleted, NAPQI may attack intracellular organelles, especially the liver, causing oxidative stress, inflammation, ATP depletion, and, eventually, apoptosis [28–30]. Our study has shown that oral NAC treatment effectively alleviated hepatic damage and reduced collagen fiber formation, oxidative stress, inflammation, and apoptosis in APAP-treated mice. The main mechanism of NAC detoxification is that NAC is converted into cysteine in the body which can be directly combined with NAPQI to detoxify. Thus, our results indicated that APAP toxicity can increase the amount of the reactive metabolite, NAPQI, while NAC can directly combine with NAPQI to detoxify. Similar studies have reported that soursop fruit extract could alleviate hepatic injury in APAP-treated mice through antioxidative, anti-inflammatory, and antiapoptotic activities [31]. Thus, identifying TCM with antioxidative, anti-inflammatory, and antiapoptotic properties that can be used to treat a hepatic injury is crucial.

## 5. Conclusions

**Figure 11:**
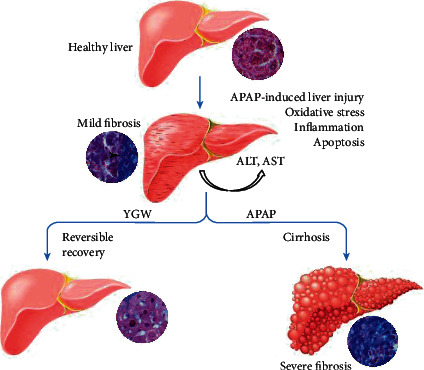
Schematic illustrating that YGW treatment could alleviate liver injury in APAP-treated mice by inhibiting oxidative stress, inflammation, and apoptosis.

The results of this study and previous studies indicated that hepatic injuries are often caused by oxidative stress, inflammation, and apoptosis. By understanding the potential molecular mechanisms of hepatic injury, we can develop TCM preparations as potential hepatoprotective medicines. As suggested in Figure 11, we found that YGW treatment exerted hepatoprotective effects on LPS-treated Kupffer cells and alleviated oxidative stress, inflammation, and apoptosis in the liver tissue of APAP-treated mice. Our results indicate the potential of YGW as a novel hepatoprotective medicine for hepatic injury.

## Data Availability

The blood biochemical analysis, ROS analysis, immunohistochemistry, and Western blot analysis data used to support the findings of this study are included in the article and are available from the corresponding author upon request.

## Abbreviations

APAP: Acetaminophen
DPPH: 1,1-Diphenyl-2- picrylhydrazyl
ALT: Alanine aminotransferase
AST: Aspartate aminotransferase
IHC: Immunohistochemistry
IL-1*β*: Interleukin-1*β*
HPLC: High-performance liquid chromatography
LPS: Lipopolysaccharide
MTT: 3-(4,5-Dimethylthiazol-2-yl)- 2,5-diphenyltetrazolium bromide
SEM: Standard error of the mean
SOD2: Superoxide dismutase 2
TNF-*α*: Tumor necrosis factor-*α*
YGW: Yang-Gan-Wan.
